# Histone deacetylase 3, not histone deacetylase 2, interacts with the major immediate early locus of human cytomegalovirus

**DOI:** 10.1186/1743-422X-8-151

**Published:** 2011-03-31

**Authors:** Ying Huang, Qiyi Tang, Michael Nguyen, Kalpana Dulal, Weijia Wang, Hua Zhu

**Affiliations:** 1 Department of Microbiology and Molecular Genetics, UMDNJ-New Jersey Medical School, 225 Warren Street, Newark, NJ, 07101, USA; 2 Department of microbiology/AIDS program, Ponce School of Medicine, 395 Zona Industrial Reparadara-2, Ponce, PR, 00716-2348, USA

## Abstract

Evidence suggests that genome chromatinization and the posttranslational modification of histones are involved in the regulation of viral gene expression, including the human cytomegalovirus (HCMV). We performed a ChIP-on-Chip assay to determine whether histone deacetylases (HDACs) interact with HCMV genomic DNA on a global level. Surprisingly, we found that HDAC3, but not HDAC2, interacts not only with the major immediate early (MIE) promoter but also with the entire MIE locus, suggesting a heterogeneous interaction of HDAC3 with HCMV DNA. The interaction of HDAC3 with the MIE region is related to inhibition of viral replication because HDAC3 inhibitors enhanced HCMV replication.

## 

Human cytomegalovirus (HCMV) is a ubiquitous virus and infects a majority of the general population (50-90%) [1]. The fact that the incidence of cytomegalic inclusion disease (CID) is intimately related to viral burden suggests that the inhibition of viral production by the specific repression of viral gene expression will reduce the occurrence of CID [2]. Understanding the mechanism of HCMV gene regulation is the pre-requisite for developing drugs that interfere with viral replication by repressing viral gene expression. The HCMV major immediate early (MIE) gene products, IE1 and IE2, are among the first *de novo*-expressed viral proteins that are required for subsequent viral gene expression and hence viral replication [3]. IE1 and IE2 mRNAs are encoded by the major IE locus that spans from 169 to175 kbp of the viral genome and are produced by alternative splicing and differential polyadenylation [4-6]. IE1 and IE2 share the first 85 amino acids [1]. MIE genes are controlled by a strong promoter/enhancer that contains many regulatory elements [3,7].

Different cellular mechanisms have been found to play roles in inhibiting viral gene expression, and one of the most prominent ones is gene silencing through viral DNA chromatinization (also called chromatin remodeling) [8], a procedure carried out by histone or histone-related proteins, such as histone acetylase (HAT) and histone deacetylase (HDAC). Several posttranslational modifications of histone proteins have been defined to be involved in chromatin remodeling, including acetylation by HAT, deacetylation by HDAC, SUMOylation by SUMO (Small Ubiquitin-like Modifier)-related pathway, deSUMOylation by SENP (a SUMO-specific protease) family enzymes, phosphorylation by kinase pathways, and methylation via methylases [9-13]. Those enzymatic pathways orchestrate to regulate cellular gene transcription and are termed as epigenetic codes [10,13]. Viral gene transcription requires cellular machinery, which is probably also regulated by cellular gene regulatory pathways. Histones are abundant nuclear proteins and have been shown to bind with HCMV genomic DNA [14]. Therefore, it was reasonable to propose that chromatin remodeling of viral DNA takes place in the nucleus, which speculation was validated when the fact that HDAC inhibitors can promote cytomegalovirus production was also confirmed [15-17].

How HCMV strips off the cellular proteins in order for the virus to replicate its own DNA is not fully understood. Recent studies have shown that HCMV and murine CMV (MCMV) major immediate early proteins, IE1 and IE2 (or IE3 for MCMV), interact with HDAC1, 2, and 3, and HDAC inhibitors enhance viral production [17-21], and dynamic chromatin modification of the MIE promoter and other viral promoters has been shown. However, interaction of HDACs with the viral genome has not been clearly demonstrated [22]. In this study, we performed chromatin immunoprecipitation followed by microarray on an HCMV DNA chip (ChIP-on-chip) assay to demonstrate the interaction of HCMV DNA with HDACs. To our surprise, we found that HDAC3, but not HDAC2, interacts specifically with the MIE locus, which suggests a heterogeneous interaction of HDAC3 with HCMV genomic DNA. In addition, we found that the interactions of HDAC3 with the MIE locus might relate to the modulation of viral replication because HDAC3 inhibitors can significantly enhance viral growth.

The chromatinization of viral DNA after its having entered the nucleus has been noted not only in latently infected viruses such as EBV and KSHV (the genomes of which are tethered to cellular chromosomes) but also in the lytic infection of HCMV [8,23,24]. On the other hand, histone proteins have not been found in herpesvirus virons [25]. Therefore, the chromatinization of HCMV DNA must be temporary and dynamic. We wonder 1) whether the HDACs are bound to the HCMV DNA, and 2) if so, where they interact and whether the interaction is homogenous or heterogeneous. In order to answer these questions, we performed a ChIP-on-chip assay.

The human foreskin fibroblast cells (HFF) were infected with HCMV at an MOI of 5. The cells were fixed at 24 hours postinfection with 1% paraformaldehyde. The chromatin immunoprecipitation (ChIP) inputs were prepared and performed using the commercial kit (EZ ChIP, Upstate Cell Signal Solutions), according to the manufacturer's protocol. The antibodies used for ChIP assays include anti-HDAC2 (clone 3F3), anti-HDAC3 (clone 3G6, Upstate USA, Inc.), and normal IgG (as a negative control).

To generate an HCMV genomic microarray for the ChIP-on-chip assay, an entire HCMV (Toledo strain) genomic DNA was subdivided into 593 small DNA fragments and amplified by PCR (primers are listed in Additional file 1, Table S1). Each PCR fragment was ~500 bp long with 100 bp overlapping the adjacent fragments. The PCR products were verified by agarose gels, purified, quantified, and printed on glass slides, as described [26]. Each DNA fragment was spotted in triplicate on each array. The printing quality of the array was controlled by hybridizing the array with a Cyanine 3-dUTP-labeled random 9-mer, and the slides were scanned using an Axon 4000A scanner at 532 nm. Figure 1 shows that all 593 HCMV genomic fragments were printed on the microarray relatively evenly.

**Figure 1:**
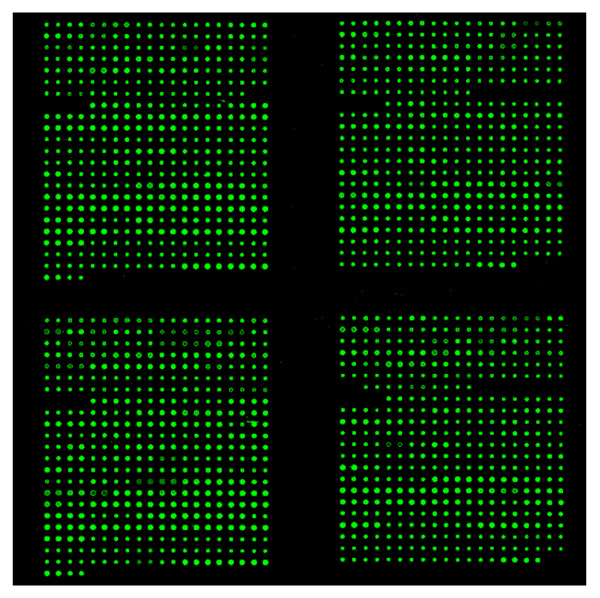
**HCMV array printing quality control**. The printing quality was checked by hybridizing the array with a Cyanine 3-dUTP-labeled random 9-mer, and the slides were scanned using an Axon 4000A scanner at 532 nm. The image showed that all 593 HCMV genomic fragments were printed on the microarray relatively evenly.

Since chromatin immunoprecipitation yields a small amount of DNA, PCR amplifications are required before labeling steps. First, precipitated input DNA was randomly primed with a sequence-tagged oligonucleotide (GTTTCCCAGTCACGATCNNNN NNNNN) to generate templates with a specific tag at both ends for subsequent PCR. Then, a specific primer (GTTTCCCAGTCACGATC) was used to amplify the templates previously generated. The final amplification consisted of additional PCR cycles to incorporate aminoallyl-dUTP. The amplified products were then labeled with Alexa Flour 647 or Alexa Flour 555 and applied to a DNA array of HCMV. The hybridization was performed according to manufacture's instruction (Corning UltraGAPS™).

The slides were scanned using an Axon 4000A scanner. Images were analyzed using GenePix Pro 5.1. A normalization factor was determined using the ratio of the median signal intensity of the cy5 and cy3 channels, and all data sets were normalized to a single medium intensity. A log2 signal intensity ratio for each fragment was calculated for the ChIP samples that were pulled down by monoclonal antibody against HDAC3 (or HDAC2 or histone H3) to that by normal IgG. To correct for the biases in the ChIP procedure, all log ratios were normalized by subtracting the average log ratio across the whole array. We considered that a spot was enriched if the signal intensity from ChIP sample of anti-HDAC3 was 4-fold greater than the corrected signal from the IgG ChIP sample.

To our surprise, we found that microarray spots corresponding to the DNA within the MIE locus interacted with HDAC3 only, not HDAC2 (Figure 2A, black arrows and data not shown). The MIE locus is only specific HDAC3-interacting region found in this study. Some non-specific hybridization signals were detected, as is indicated by the grey arrows.

**Figure 2:**
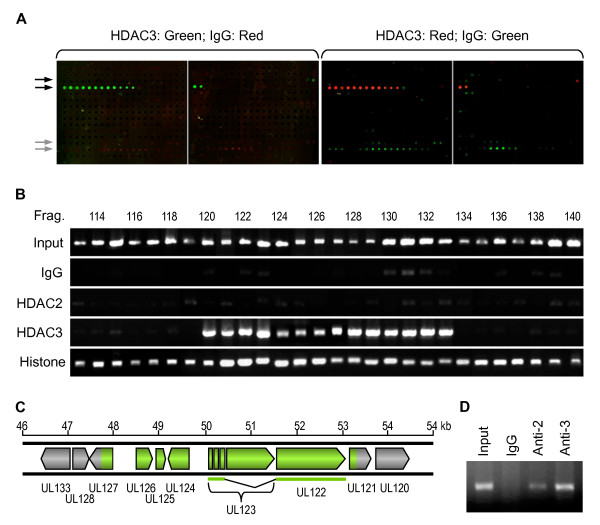
**HDAC3 but not HDAC2 interacts with the MIE locus of HCMV**. **A. ChIP on Chip assay**. HFF cells were infected with the Toledo strain at MOI 5, and the DNA samples from the infected cells were prepared by using the EZ-ChIP commercial kit. Anti-HDAC2, anti-HDAC3, and normal IgG (as a negative control) antibodies were used to precipitate the fragmented Protein-DNA complexes. The DNA fragments pulled down with HDAC3 and IgG antibody were amplified and labeled with Alexa Flour 555 and Alexa Flour 647, respectively and vice-versa. The labeled DNA was applied to the HCMV genomic Microarray containing the entire HCMV (Toledo strain) genomic DNA divided into 593 small fragments. The array was scanned at 532 nm (for Cy3) and 635 nm (for Cy5). HDAC3 was detected only in the MIE locus as indicated with black arrows. The grey arrows indicate no-specific hybridizations. **B**. PCR verification of the ChIP-on-chip assay. A series of PCR reactions covering the MIE region was performed on the sample before ChIP (Input) and ChIP samples, as indicated on left. **C**. HDAC3-interacting map. The ORFs spanning the MIE locus are shown. HDAC3-interacting region is indicated as green. **D**. HDAC2/3 ChIP on hTERT promoter. Mrc-5 cells were cross-linked and ChIP samples were made using the EZ-ChIP commercial kit. Anti-HDAC2 (Anti-2), anti-HDAC3 (Anti-3), and normal IgG (as a negative control) antibodies were used to precipitate the fragmented Protein-DNA complexes. PCR was performed to detect the precipitated hTERT promoter DNA using the primers and PCR protocol as reported [27].

To further confirm the ChIP-on-chip results, we performed a series PCR reactions using primers amplifying HCMV genomic fragments 113 to 140 (Figure 2, Additional file 1, Table S1). As shown in Figure 2B, all HCMV genomic fragments were present in the ChIP input sample; IgG and HDAC2 did not specifically interact with the HCMV genome; histone H3 interacted with the entire viral DNA tested; and most importantly, HDAC3 interacted with fragment 47-53 kb of the Toledo genome corresponding to the HCMV MIE locus (Figure 2C, green). To confirm that the anti-HDAC2 antibody is effective for the ChIP assay, we performed another ChIP assay using anti-HDAC2, -HDAC3 antibodies to bind ChIP samples made from uninfected Mrc-5 cells. The eluted DNA samples were applied for running PCR against hTERT promoter as reported by Straat et al. As shown in Figure 2D, HDAC2 bound to hTERT promoter, which is consistent with the report [27].

Since HDAC3, like HDAC1 and 2, is one of the major components of nuclear co-repressors (DNA-remodeling complexes) inhibiting gene expression, HDAC inhibitors should be able to counter HDAC3's repressive effects on gene expression so that they can promote HCMV viral replication. To confirm our speculations, we infected HFF cells with HCMV (at MOIs of 0.1 and 5) in either the presence or absence of the HDAC inhibitor, Trichostatin A (TSA). Growth-curve analysis showed that the HDAC inhibitor significantly accelerated viral replication (Figure 3).

**Figure 3:**
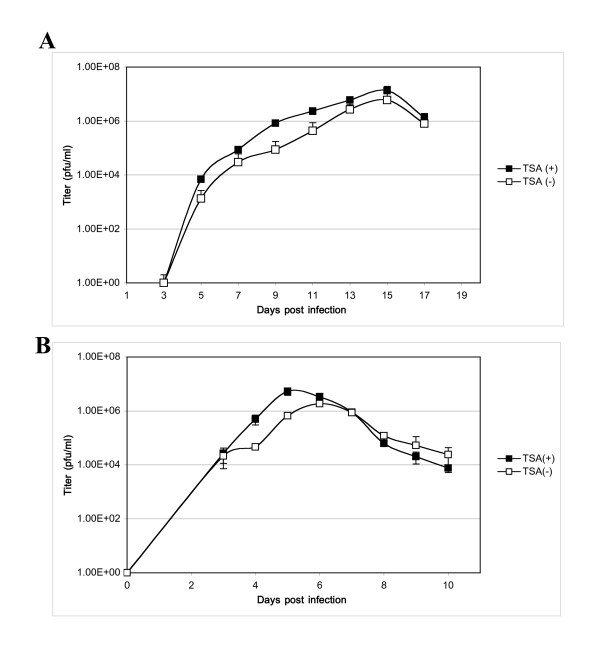
**HDAC inhibitors enhance viral replication**. HFF cells were infected with the AD169 strain of HCMV at MOI = 0.1 (panel A) and MOI = 5 (panel B) both in the presence and absence of TSA (250 nM). At different times postinfection, virus titer was determined using the plaque formation assay. Data are expressed as the average of three independent experiments. As seen in panel A and B, the production of infectious viral particles with TSA is approximately 10-fold higher at its maximum than without TSA.

We demonstrated that HDAC3 but not HDAC2 interacts with the HCMV MIE locus. The reason that HDAC2 was not detected in our assay could be due to the fact that HDAC2 is known to interact with IE2, and this binding suppresses the repressive effect of HDAC2 [20]. At 24 hrs postinfection, the genomic region around the MIE may need to stay repressed, which could be accomplished by the action of HDAC3. This binding of HDAC3 to not only the MIE locus but also the genes upstream of MIE is intriguing. The region between the MIE and UL127 has been named as a unique region without any known function. This region is known to have binding sites for various cellular repressor proteins that help to repress the transcription of UL127. It is known that the promoter of UL127 gene is silenced during productive infection in fibroblasts [28]. The UL126 gene has been reported as a latency-associated gene [29]. UL124 is a putative membrane glycoprotein that may be expressed late in the infection process [30]. It will be interesting to see how the HDACs bind with those genomic regions at later times of infection.

HDAC3 has been shown to be a repressor of the viral MIE promoter [14]. A significant increase in viral replication in the presence of the HDAC inhibitor demonstrates that the binding of HDAC3 causes inhibition of viral replication. This inhibition seems to be more pronounced at a low MOI. Host cells contain several proteins including gene expression suppressors that form a defensive arm against viral infection [31]. HCMV has evolved strategies against cellular defense. We speculate that HCMV infection must reduce HDAC activity. Both IE1 and IE2 of HCMV were reported to functionally interact with HDACs [14,18-20], and IE1 of MCMV is particularly adept at reducing HDAC activity [17]. These observations and our finding of an increase in viral replication in the presence of TSA further confirm the IE2-mediated repression of the MIE promoter by the recruitment of chromatin remodeling factors.

## Abbreviations

HCMV: (human cytomegalovirus); HDACs: (histone deacetylases); MIE: (major immediate early); CID: (cytomegalic inclusion disease); SUMO: (small ubiquitin-like modifier); SENP: (SUMO-specific protease); HFF: (human foreskin fibroblast cells); TSA: (Trichostatin A).

## Competing interests

The authors declare that they have no competing interests.

## Authors' contributions

YH developed HCMV genomic microarray, carried out growth curve, PCR and data analyses QT carried out ChIP-on-Chip assay and drafted the manuscript. MN developed HCMV genomic microarray and carried out ChIP-on-Chip assay. KD carried out growth curve analysis. WW participated initial studies and carried out growth curve analysis. HZ conceived of the study, and participated in its design, coordination and manuscript preparation. All authors read and approved the final manuscript.

## Supplementary Material

Additional file 1 **Table S1**. PCR primer sequences for HCMV genomic microarray construction Click here for file
