# Supplementary material for: Histone deacetylase 3, not histone deacetylase 2, interacts with the major immediate early locus of human cytomegalovirus
Source: Virol J. 2011 Mar 31;8:151. doi: 10.1186/1743-422X-8-151 (PMC3077330; doi:10.1186/1743-422X-8-151)
Supplement: Additional file 1 — Table S1. PCR primer sequences for HCMV genomic microarray construction [file 1743-422X-8-151-S1.PDF]

Table S1

Oligo pa:	Start	End	Forward strand	Reverse strand	Size
1	1	500	GGCGATGAAGAGCCAGAGTATGG	CGCCGCCGTCACCACCGCCGATC	500
2	400	900	GTAGAGACGTACCATCCAGAGAA	AGCGTATTCTACTCTACGGCTAC	500
3	800	1300	CTGAGCAGCCGGCAGTTCGTCGC	ATCAGTACGGCTTTCTGCGGAAT	500
4	1200	1700	GAGGGGGCAAAAACCAGCAGCAG	TGGTACTTTACGCCACCGAGACA	500
5	1600	2100	CAGGTAGCTGAACATTTGGTCCC	TTGGTTCTGGGCGCGCTGGAGCT	500
6	2000	2500	AGCCCGGCGGTGGTGAACGAGAG	ACTTTTCCTCTGACAAGCTGTAG	500
7	2400	2900	ACGCGGTTTCCCGCTGCGTGGAA	ATCGTGATATGTCTCTGCCTGGA	500
8	2800	3300	GGAGCCAACAACGCTACGCCGGT	ACAGAGCAGCCGTTTCCAGAGAA	500
9	3200	3700	GGATCCCGGGGGGCACGTAGATG	ACCCTCATCAACGGCGTCTGGGT	500
10	3600	4100	TAGAAGCCCAAAAGCAGCCGCAG	CCTGTTTCAAATCTCCGCGGCGG	500
11	4000	4500	CAACGTGACAAGGAAAGCCCGGA	GGTTATCGTCACGCGCCACCGGG	500
12	4400	4900	TTACGCCATGGTTCGCGTGAGGT	AGCGCCTGCTCCTCCTCTACGCT	500
13	4800	5300	TGATTATGGGCGGCTAGACCCGT	TTTGATCGCCACCGCCGTCATGT	500
14	5200	5700	AGGAAATCATAATGACTCCGCGC	ACAGCGTCTGTGTCAGCGTCGTG	500
15	5600	6100	GGCGTACTGCTGCACCCAGACGT	CTGGCTATGCTACGCGGTTTCGC	500
16	6000	6500	CACACGGTGATGCCGGTGTTGAT	GATACAGGGTGCCGCCACGGTTT	500
17	6400	6900	TAAGGTAGGCGTCGATGAAACAG	ACCTTGCTGATGTGCCAGCCGTC	500
18	6800	7300	CAACCCAAAAGCGAGGCCCCCAG	AGTACGACGGGGACGTCCTAATG	500
19	7200	7700	CGACGGTCCATTCTAGCGGGACG	AGTAGGTTCCTACGTGATGACCC	500
20	7600	8100	CGCAGCTGATGCATTTCCAACGA	TGTATAAGAGAAGGGTAGGTGCG	500
21	8000	8500	CTCCATGCGGGAGAGCAGCAGCG	GCGACGTGGAATTACAGCGTTCC	500
22	8400	8900	GATCTCACTTACCGCCTCGGGAA	ACCGCCGGCGTTGCCGCCATGTC	500
23	8800	9300	ATGAGGCATTGCACCCAGATAAG	AAACCCCGTGTAATAGCGAAAAC	500
24	9200	9700	GTCTTCCGACGGGTAGGGAGTCT	GGCGCACTGGGCACCATTACACG	500
25	9600	10100	GCGCTGCTGTTGTTGGGTACCTT	GGTGGAGCCGTTGCTCATGCTGG	500
26	10000	10500	TAAAGTTCAGAAGATCGCGAAAG	GTTCTCTGGCCGCCGACTATCTC	500
27	10400	10900	CTTTGTGTTTCGTGTTAGATCGC	GGCGCGTCCCACCCCCCGGGCCA	500
28	10800	11300	CCGTCTCGACAGCGAGTCGGATA	CGGACGATGAGGAGCAGGGAGAG	500
29	11200	11700	TTCCCCTCCTCTTTCAACGTTGG	GGTCTGCGGCGCGCGGCCAGGCT	500
30	11600	12100	CGGTCTACGCCGCTGTCGAACAT	ACACCACGGACGCGACGTGAACT	500
31	12000	12500	CGAAGGGCCAGCAGTCCGTCAAG	TGCTGTCGCTCGGCATTCCGCAC	500
32	12400	12900	GCAACAAATCACGTCGTCCAGCA	TTCGTCGACGCGCGAGGGCGCGG	500
33	12800	13300	CCGTCAGGCTCTTTAAGGGAAAA	TGAAAATGGTCTTTCGACACCGG	500

34	13200	13700	TACGGCACGCTCGTGGTGATGCG	ACCATGCATGTTTTGTTTCTCAA	500
35	13600	14100	TAACCCGGTGTATGCAACTTCAC	CGAGGGACGCTGTCTGTCCTTGG	500
36	14000	14500	GCTTTGACGCCGCCTGTTTGGCG	TGTGAACCGCTCAGTGGCTCGGA	500
37	14400	14900	CCGTCGTTCGCGACGTTGATTTG	CAGTTTACGAGTACAAAACAGGA	500
38	14800	15300	CAAAAAAGAGCACGTGCCGATGA	CCGTCAGAACCTGATGAATCCCA	500
39	15200	15700	CATCGTCGGACGTGTCGTCTTCC	CCGAACGTCTGGAAGTGCCGGGT	500
40	15600	16100	GTGATGGCGTGTAGAGGCCCGTA	CTACAGCGTGTGACCTGTCGTTA	500
41	16000	16500	AGGTTCTCGGCCTGGCCGCAGAG	CGAAACGCCCTGGGGTCAGCTCA	500
42	16400	16900	GCGGCCCGTGTACTCTGGGCAAC	CACTTTTCGGTCGCACATGTTCC	500
43	16800	17300	TCGTGGGCTGCGGTTGGCAGCTC	AGGTATCGCTCGGAGATTTTTTC	500
44	17200	17700	CGGGGTATGGTTGATGTGCATCG	AGGTAGCCGCAAATGTTGACTTT	500
45	17600	18100	TACAGTCATACCAATAACGAGTG	TTGTATGTCCTTTTGTAGCTCAA	500
46	18000	18500	GGAAGGACATGTGGACAACCCTA	ACGTAACCGGCTTTGATTGATTA	500
47	18400	18900	GACGACGACGACCGCGGAACTCA	TTTTTTTTGGTCACCACCATAAA	500
48	18800	19300	AGATATCGGCCTGTAAAACAGGC	GTGCAGTTCTTGCCGAAACTTGG	500
49	19201	19690	ACGTGCGCTCATCTTGACCGAGT	CGTCACGGTCCGAGCACATCCAA	489
50	19618	20096	CATGCGGTGTTTCCGATGGTGGC	TCACGTACGCACGATTACCCCCG	478
51	20007	20496	AGCGCTGGACACCTGTTCGGAGA	GCCACGCGTCGCCAGAAAAGCAG	489
52	20394	20913	GTGTGGATCTTCTGGTGTGCGTC	GAGACCGCGCCGTCGTTGTTGAC	519
53	20797	21294	GGCGCACGCTACTTATGGGGAAC	CGTACTTGGTGTCGCCGGTTTGT	497
54	21179	21700	TCATCTCAGCGGACTCGGGTGTC	TGCAGTTTGCCTTCAGAAACGGC	521
55	21587	22097	CCGGGACAAACATCACGCATCGC	CTCGCAGTTGGGCTCCTCGTCCT	510
56	21994	22500	AAGAGAGTTGGCGTCGCGTAGTC	TCCGGTTGATGTAGCCAGTCGCC	506
57	22396	22900	GCGGTGACGAGAGTAACTGCAGT	TTCCAAGCGGGGACCTGTTGCTC	504
58	22790	23300	GGACTGGAAGTGTCACGCGTTAT	ACCCGCTCACCACCCCGTTCTTA	510
59	23201	23710	TGTCCCCATCATCAGCGTCACAA	AGCAGACCAAGGACGTTGATGAG	509
60	23609	24118	ACATAAAGCCAAAGCGCCGTCGG	GACGCTCCGTGAAAATCACCGAC	509
61	23995	24512	TCCTCCTCTTGAACCGCTGTCAC	AGAAATGAAAAACCAGGACACGC	517
62	24391	24900	CGGCGTGTGTCTTACTGACGAAG	ATGAACACCGCCACCAAGCCTCC	509
63	24797	25297	CAGCTGTATTGTGCTAGCCCCAC	ACAACACTATCACAACGCCACGG	500
64	25200	25700	TACAGGCTACCGCGTGGTGTTGT	GGACGAGACTTTCTGGGTTCTGG	500
65	25592	26081	TGGCATGGGACGATCAATCTCCA	CGAGCCGCACGGGCAGTTAGCTT	489
66	26000	26515	CGGGTGTTTGGTGGCTAGGGTGA	GTACCTGTGCCTCCGCAGCCATA	515
67	26374	26881	TCGCCGTCGGACCCACCAACACC	GCAGGCACGACGGTGGCACTACA	507
68	26791	27307	GTTCGTACTGCGCCAGCTCCTGC	GTGACGCCGCGCAGCCAGATGAA	516
69	27210	27686	TGCACCACGGTCGCCACCAACAC	AGGGCCTTCGGGGGTTGGTT	476

70	27596	28103	ACCCGCAGTACTCGCACCAGTCC	CTAAAAACACCCCCCCGCCCCTC	507
71	28001	28481	GCAAAGCAAAGCCGCCTCCCGAG	GCGGCCGCTTCTGCGTGTGTCCT	480
72	28396	28908	AAGAGGAAGCGCCTAGGGGACCG	CACCGCAAACCTCCCTTAGTCCG	512
73	28795	29304	ACAGGGCTCACGGAGGTTTGCGG	CAACCCTCGTCGCTGCAAAAGCC	509
74	29207	29707	GCGAGGACGGGGGCCTTTTG	GCGTATGTTCTCTCGTGGAGGCG	500
75	29603	30103	CATCCTACGTCGTCTGCTCACAA	GTGTCCTGCAGATAGTTCCACGG	500
76	30000	30493	ACCACAAACTCTGCGGCGCGATG	CGAGCTACGCAGACGGAATATC	493
77	30387	30913	CAACAGGCCGCACACGTGTCGAC	GCGTAGCTCGCCGTCGCTAT	526
78	30798	31305	CCAAAGCACACACGCAGCAGCAG	GTGCCTAACGCGGTATACCACAC	507
79	31201	31700	GGAGCGACAAGACAGGAACGTGG	CTTCTGCTCTTCCTCCTTCACCG	499
80	31589	32100	GACACACTTGGGCGCCGTCATGC	GGATGCTGGTCGTTGGCCTGATG	511
81	32004	32505	GTAAGCACGGGGAAGGAGCGCTG	CGGCAGGATAGCGGTTAAGGAT	501
82	32402	32905	ATTGCGATGAGAGAGGGGATGGC	ACACTTCCACCATCTCCAGCTCG	503
83	32812	33300	GAGAAGGTGTGCGTCCGCTGCTG	GCGTTCTGTGGTGCGTCTGGATT	488
84	33192	33700	ACCGCAGCCTGTGGATTCATGAA	GCTGCTACGCTTCGTCGTCAACG	508
85	33600	34105	GAAAAGACACGCTGTAGTCCCGG	CTGTCTGGCGTCTCCGTGGTCGA	505
86	33996	34502	AAGGACAGTAAAACGTCGCCGCG	TACCACTACGATGCGAGCCACGG	506
87	34400	34916	ACCCGTTCGCCCTTACCTTCCCG	GTGTACGATGAACTGACCGCCTC	516
88	34797	35302	GTCGTACTCGGGATCTCTGAGCG	TCGCGGTAGTTTACTCCTCGTGT	505
89	35206	35693	CCACTGTCGTCCGTGTCGTCCAA	GGCGTCGGTAGAGGCTTGCGGAA	487
90	35600	36101	CCGGCATTCTCGAGATGTGGCGA	TAGCTCTGGCGCCCGATTTAGTA	501
91	36000	36500	GGTAGTGGGTCTGACTGCGACGC	GTGTGCGTCGACCCTTCACCGAG	500
92	36406	36890	CTGCTCCAGATCGGTAGACCAGA	AGCGCTTTGGTGTGGTACAGCAC	484
93	36800	37310	CCTCGCAGTCTTCGTGATCCGCA	TCCCCTAGCCGTCCATGTTTTAG	510
94	37191	37700	CTCGCTGCGATTGGCTTAAGCAA	TATTGGCTTCATCGCGATCCCAG	509
95	37595	38100	CGGGCGATAAACATACTCCAGGT	CCTGACAAGAAAGACGAGAGAG	505
96	37994	38500	TCGATCACACGTCAATTAGG	GTACGGCGTCCTGGCTCATTACT	506
97	38398	38889	TCGGGTCCGTGCACATAACAGCT	AGCGGTCGTCATACTCTAGCCTG	491
98	38787	39317	GGCATCTCTCACTCCGATAGGCG	ACCGTTCGGCCCCATGAGTTATT	530
99	39208	39697	CACTTTAGTCACTCCAAGCTGC	ATTGTGCTCCATCTGGGTAACCG	489
100	39603	40102	GTGTGCTGCTGTGATTGGAGGCT	CGACACACATAACGTGACGA	499
101	39992	40522	GCTGTAGCGCCCATAGGTATG	CGAAAACATTCATCGCGTATCTC	530
102	40421	40918	GTATAGGTAGGTGTACTGTTTG	GATGAAGAGGTGAGAACACG	497
103	40809	41298	ACCACCACACCCATTCAATCCGC	AACTACTTCTATATCGGCCGCGC	489
104	41196	41695	GCCGGTAACGCTGTATCACTGTC	GGTCTCCATTCGCGCACGTTTCT	499
105	41599	42101	CTGGAGTCCGTTCATCAGCCGAT	GAGGGGGAGGCAGCAAAAAAGAC	502

106	42014	42500	TGACCACCGTGCGACGCAGCTTC	CGTTCTTTTATCACCCGCGTCCG	486
107	42395	42884	CGTGGCGTAGAAAACCGAGGTGG	TAAAACACCGTAGAGGCTAAGGC	489
108	42804	43300	CTGAAGAGATGAGAGCACGGGTG	CTAGTGCTACCGCCAACACGACC	496
109	43190	43701	CAGATAATGTCCAGCCGCTAGCG	GGTGGCGGTGATCGGTGTTAGAA	511
110	43600	44099	CGAACTCATGTGAAAAGTGTGC	TCCACGTCGCTAGCCAGAGAAAC	499
111	44008	44499	CGTCACGGGTTTCAACAGATCGA	AGAGAGAGACTGGGACGTAGATC	491
112	44398	44900	GATCAGCACGAGCATCACGCCGA	GAGAAGGAGACGGCAAGCCATGG	502
113	44803	45304	GACAGCCGCTGCCGTAGTACATA	CCTTCCCTCTTTTTCACCGCAGC	501
114	45186	45698	GCCTTTCGACGCCGCCATTTATT	TAAGAACCTGAGCACGCCGCCCA	512
115	45600	46094	TTCGGGAAGGGCCTGGGTGTCAC	GAAAGAAGACGTCGTCGAGCCGC	494
116	45996	46502	TAGAGCGACGTACACCGCGGCAC	TGGCCGCGGCTCTACAACAACAG	506
117	46400	46910	ATTGAGGAAGGAGATGTGCGCCA	CAGCGTGTTACAAGGAGACGATG	510
118	46793	47295	ACCGGGGGACTCTCGACTATCTC	GTATTCGCCCGTCAGCCGCTGTA	502
119	47204	47706	GAAGGATCGTGCACGTCGCAACC	GCCAGATATCGCGTATGCCCAA	502
120	47615	48100	GGCATATTGAAAATGTCGCCG	AACGCGGAACTCCATATATGGGC	485
121	47993	48486	GGCTCATGTCCAACATTACCGCC	TTGACGTCAATGGGGTGGAGACT	493
122	48400	48914	GGTGATGCGGTTTTGGCAGTACA	TATCGTCACCAATAGGGGAGTGG	514
123	48814	49289	CCTATACACCCCCGCTTCCTTAC	GGCACACTGGTGGTGGTGGGCAT	475
124	49208	49685	CATGGTCGCTCGGCAGCTCCTTG	AAAAAGAGAGGGGCACAAACC	477
125	49602	50101	GAAAGATGGACCCTGACAACCCT	AGTATCCGTCCTCCTGACTCA	499
126	49993	50514	CCTGCATGGGACGTGGATTTACT	GAACTCTACATTCCTATAGCAC	521
127	50400	50908	TGTGACCAAGGGTGCAGCTAACA	TCTCCTCAATGCGGCGCTTCATG	508
128	50793	51303	GTTCTGTCGAGTGCTGTGCTGTT	GCCTCCAGAGGTGGTGGGTTCCT	510
129	51191	51692	AGGAGGAGCGGGAGGACACTGTG	CGGTTTCTTACGCGGGCTGAGCT	501
130	51601	52100	TTAACAAGCCGACCCCCACCAGC	TCCGCCACTGCTGCATTTCATCT	499
131	52001	52494	CCGGCCCGATGAAGATAGTTCCT	TGCGGGTGTAGATGATCTGGATG	493
132	52400	52912	GATTCCCAGTATGCACCAGGTGT	GTCTCGGTGGGCTGCTCAAACTG	512
133	52799	53302	CAAACAGGTCATGGTGCGCATCT	TGAGACGACCCGATGAATACTGG	503
134	53198	53700	CTTACATCTGCTCCCCCAACCCA	TCTCGACGTCGCCGGCTGTCAAT	502
135	53596	54118	GCCCTGTGGAAGGTAGATTACGA	CCGGCCGTCGTATTGATCATGGA	522
136	53994	54519	CTGAAACGCATGCTGACAGGGGC	TGGTGGTGACGGTGGAAGTAACG	525
137	54398	54916	GAGTAACACGCACCCGGGAATGG	TGAGCCGTGGCATTGTAGGACAG	518
138	54806	55300	CCTACCTTTCCGTCTATTCCCCT	GTGGTGGTAAAACTTCGGCCCGC	494
139	55203	55700	AACGGACAACGGTTACGCGAGTT	GTCTTTCTCTTGTTGGCCGGGGC	497
140	55600	56101	GCGTTGGCGGCGATGGTCATGTT	GACAGACTTCCACAGTGGCGGGC	501
141	55996	56483	CCTGCGGCCATGGATCATAGTGA	CTGATCTGCAGCTGCACGGCCGT	487

142	56385	56890	GTACGCGCCACTCTTTGCCAACA	TTCATGAGGTGGGCAGGCGAAAG	505
143	56811	57307	ATTGACGTACCCTTCGATCGCGG	GGAGCGCGGGTGGTGGTAATGTT	496
144	57196	57697	CCATACTTACCACCGTGACACCG	ATTGCATCCAGCAGGTGAGCCAG	501
145	57600	58100	CTCTCCAACTGTAGCGCCCACGG	AAGCATCGTCCAACAGCACGGAG	500
146	57989	58500	TGGCTGCGTCCGTTGGTAAATGT	AGCTGGTGACGCAGGCAGAATTC	511
147	58378	58880	CGAAGCCACGCGTACCAACC	GTCGCAGTACGGGTCCTGGC	502
148	58802	59307	GCACGTACACCGCTGGAGCTATC	TGTCCAGATACGCGTTGGCCAAT	505
149	59202	59697	CGACGGCGGCACTTGGTACTCAA	AGAAACAACCGCCACGAGGACGA	495
150	59590	60100	GAGTCCGTCCTCGAGGAACGA	CAACCACAGCAGCGTCGCGAGGA	510
151	60010	60508	GGTGGTGACGAGTGGCGGACATG	GGTGGTTTCGTCGCCGTCGTCGA	498
152	60410	60893	TCGGACTCCTGCACCGTCTC	GGTGTTGGTGCGCTGGTGGTTGT	483
153	60804	61314	GGAGGGGAGGAGAGCCTACAGAC	AAAACCACGGCGAATCCTCCAGC	510
154	61191	61717	TGTTCCTCCGCAGAAGGGCCGTC	ACCGTCGTGCGAAAATACTGGAC	526
155	61602	62105	CAGTTGACCAGACGCGCGGTATC	AGCCTCTGTTAGGTTGCGGAGAT	503
156	61998	62500	ACGTGTCCAACTCGCTGAGACCT	TCTCCGCGTCACCTTTCATCGAG	502
157	62394	62910	GAGGAAAACTACGTCACCCGACA	ATTTCGTCTTGATCTCCAGCCGG	516
158	62792	63303	ACGGGTTATCGTCACCACTTG	CTATGAAGGTGCTATTCTTTG	511
159	63203	63696	ACACTCATAGAGAATCACAG	GCTTCTCCGTAACTATTTCCGC	493
160	63608	64101	CACGAACCAAAAGCCACAGACCC	TATCCTGTTCTATGGGGTAG	493
161	63994	64500	CTGTGTGGTCTCCGCGATCATGA	ACGTCGTTGCTGCTGCTGCTCTT	506
162	64403	64880	GCAGCCACTCTAACCATCACACT	CTATGTAACTAGGATATGGTCC	477
163	64798	65283	TGAACTCTTGCTCGTAACCGCCA	CTTGGAAAAAAAGAACGGGC	485
164	65203	65714	CAACCATGAATGATAGCCGCC	CCGAGTCGAGTTACAAGTCAC	511
165	65601	66101	GTTGGGAGGTAGCTTCGTCATGT	CGGTACGGGTTCTTGTTATGG	500
166	66021	66510	TTTGAAACGAAGATAGGCCT	GTGAGGAGATGTTCGATGATCG	489
167	66382	66889	GATGAATCCTCGATATACGCC	GCCAACAGCTATAATTTCTCTC	507
168	66790	67287	CGGTTCCCTCTCTCTAATTCCCT	ACTTATCCGTTTCCCTGGTCCGC	497
169	67203	67684	CACCCGGGTTTCTTCTCTTGCAG	CATCTACGTGGCCATGTCGCGAG	481
170	67600	68100	GTTCTTCTCATAGGGCAGTCGCA	ACCTACAATCGCCGTAACGTGGT	500
171	67997	68517	TACGTCTGCGCCGGCGACACATG	GTTGAGCTGCCCGACGTGAGTTC	520
172	68400	68904	CCTGAGAAGAATCCGCGTCGACG	GCCACCAACAACTCCTCCGTCCA	504
173	68797	69318	GGTGGTGGTATCATCGTCCTGGA	GGCGACCTGCTCAAGTACATGGA	521
174	69207	69727	CGGCGTACGATGGGTTGCGGATG	AAATATGTGGGAGCGCAAGGCCG	520
175	69600	70099	GCATGTAGCGCAGCATAAGGCCG	CTGCGTCGACCGTTGGCCCTTCT	499
176	69995	70492	TCTAGATTGGCCGCCAGCACCTG	CACCTGGTGCATGCTTTGCCCGC	497
177	70395	70921	CCGTCATCGACATTTTCCCCACG	CAGCAGCAGCGCCAGGTTAGCCT	526

178	70797	71317	ACGTGAAGACCAAGCCGAGCGAG	GCGCGATTTGCTGTCCGAGACGT	520
179	71200	71718	CCGCCACGAGCTCAACTGCAAGA	GGCAGGTGGTAGAGTTCGGCGTC	518
180	71598	72103	CGCTTAACCAGACCTGGGAGAAT	GCAAATCAGCTCCACGTATCGGC	505
181	72012	72498	TACCCGAGGCCTACCTGCAGCTC	CGGTCTCGAGCAAAAACACGGCG	486
182	72405	72908	CACGGGCTGCTGGTTTCCATGGC	CTTACGCTCGGTCAGCTTGAGGC	503
183	72800	73311	CTTCCAGCAGACGCGGCATACCT	CTTCGTCGGCGACTTGGCTCGAG	511
184	73184	73700	GACTCGTTAAGCGTTGAGCCGGA	CGTGTACCGCGAGATCCTCTTTC	516
185	73600	74104	CGTGCGGCCTTACTTCGTTTTCA	GTGCTGTTGGTCTGGGGCGACGA	504
186	73986	74501	CAATGTCCCGCCGCGAAACCACC	AGTGCTCATCGTGGATTTGGTCG	515
187	74398	74890	CCGCATAAGTGTGGAGTCGATGA	GTCTACGCGGTCCACGGTCTGCA	492
188	74812	75284	CGCACCACGAGAGCACCTCA	GGAACGCGTCTCCGAAGAGT	472
189	75191	75697	GACGCGAAACGACATGGCCAAAT	CTGTTTTTGGATCTGGGTGGCGG	506
190	75599	76104	ATGCACAGCACGCACCTCAGCCG	TGCGGAAAATTGCTCAGCACCAG	505
191	76013	76516	GTGAATACACGGACATGGAGCGC	GCGGTACTGCGTGACCATGCTCA	503
192	76417	76902	GACCATGTCATTTCGGCTGCCCA	ATGCCGAACGACCAGCTGATGCC	485
193	76800	77318	TGGGCGCCTTCTTTGGACTCTGC	CCGTGGGTGTTTCTCCGTGTGTA	518
194	77217	77722	CTTCCACCAGCTACTTTACGTG	ACGACAGTAGTAGCGGCAGCCAC	505
195	77612	78119	TTGACTGCTTGCTCGGTTTCGGA	GGAAAGCGAACTGGGCTGCGAGT	507
196	78002	78500	GGCATGGCGCGTAAACTGAGGGT	TGACGCCAAGCACCTGAATGACG	498
197	78379	78885	TGCCCCGCAGGTGGTGATCA	GCGCTTCCATGGACCTGTGCTTC	506
198	78796	79274	TGATCTCGTAGACGCGCGCGCAC	CGGCTCGGACGACTACGTGT	478
199	79196	79700	CAACTGGAAGAGCTCGACCTGTC	GAGGGGAAAGGTGACAGAGAAGG	504
200	79600	80114	CGTCGTCGTAGTCCAAACTCGAG	CACGGAGGCGTTGCTCTTTAAGC	514
201	80000	80511	GTAGCTCATTTGCGCCGCCAGAA	ATCACCAGTGTCGTGTATGCCAC	511
202	80393	80916	CTCGCTGAGGCTGTAATCGCACA	TCTACGTGCCCAAAGAGGACGAT	523
203	80799	81314	TAGCGATCGAGCGGCCAGACCTC	CAGCGACCCCTCAGACAGCGTGA	515
204	81182	81679	TTCTTCGAGCACCGCGTCCACG	GCTCAGGTTGCCCAGGCTCACGT	497
205	81589	82093	GAGTGGTCAACGTGGTGACGCCA	GTGGACGACGCGCTGGACTCTTT	504
206	82000	82505	AATCGCACGTCCCCGAAACCGAG	TTATCCAAGACTGCGCCGCGGCT	505
207	82400	82907	AGCAACTTGCCGACGTGGTAGAC	CCGTGCGGGACTATATGACCTAC	507
208	82783	83300	ACCACTACCACCACCACCACCCG	CCGCCACGCCGTCTTTTCTACGT	517
209	83182	83706	GGTAACACGGTCAGCATTGCGTA	CCCTCAACTGCCGTCAGGTCTCC	524
210	83619	84094	ACTGCGCCGACAGCTCGACCATC	GAGTTCGGGGTGCATCATCTGGT	475
211	83997	84500	AGAAGCGCAAGAGCCACCCCACC	GGTACTGCAGCACGCGATTGGCC	503
212	84398	84900	GCCATCCGACTCGAACCGTTTCA	AAGAAAGGAGGACATGGACTCGC	502
213	84792	85303	CCAAGAGCACGGCGCTTTTTGC	TTGCACGGATCACGAGGGACACG	511

214	85211	85708	CCACAGGGCACGTAACAAAGACA	TCGTCGACGCATCTGTCATCTAC	497
215	85596	86116	TCATACTGCTGGTCACCTGGCTA	ATCGTTCGTGATCGCCGCGGAC	520
216	85994	86510	CGAGAACACAGCAAAGCCGCCCG	GCGCGACGAGATCTATGCTGTAC	516
217	86400	86898	CGGCAAAGTTCCCAAACAACGCG	TCAGTAACCTGGAGCAGCGGGTA	498
218	86797	87302	CGTGACCGCGTATCTTGGAAGGC	ACGTCAAAGCGGTCTCACTGTGA	505
219	87189	87700	TGCGCGCCTGAGAGCCGATTATC	CCAAGTACGACGCGTGTGTCATC	511
220	87594	88100	GCCAGATCCGAATACAGGTGCGT	TGCTTTCGCACGCACCTGTGTGA	506
221	88005	88499	CTCGTATACCAGGCCCGTCTTGA	CATGAACTCGTTGCTGGCGGAAC	494
222	88405	88900	AGTGTTGAAAGAGGCGGTCGAC	AGGCGCGTCAAGAAACACGTGGC	495
223	88797	89307	GCCTTCAACACCATTCTGGGGTT	GAAACTGGTGTCGGTAGGCGCCT	510
224	89179	89699	CACTCAACACCAACGCCTACGAT	CTCGATGGGGTCGTGGCTCAGCT	520
225	89600	90083	GGCCGTAGAACAGTTCATCTCGC	CGAGGAGAACGACCTTGTCAACG	483
226	89974	90500	CGGCGTAGTCGGCTAACAAAG	TCTAGCGCCGTTGTTGCAGAGTG	526
227	90418	90893	GCAAACGGTGATAAGCGCTGGCC	GAAGCATAGCGATCTGGTGTACG	475
228	90805	91287	GCCTACTACTCCCCAACTGACCG	ACATCGGGCCTGTGTTCATC	482
229	91204	91709	AACGCTGACGGAGGTTGGGACAG	CCTTCCTGGCCAAGCAGTTAGTG	505
230	91600	92110	CCGCTGTGCCGCCTCAAATACTA	GCTCAACGTCAATATTCGCGG	510
231	92006	92500	CACACCACGGCCGGCAGAAAGAA	AGAGACGGTGGAATCGGGGGAGA	494
232	92395	92909	GGCTAGATGTTTACGACGTCGGT	GTCAGGGACACCACAACCTACTG	514
233	92793	93313	ACCCCAGATCCCGAGCTAGTTCA	CGACTACGTAACCGCCGTCTCCG	520
234	93211	93719	CGCTTGCGGACGCACGTTGAGTC	TACTGGGTCGTCTGTTGCTGGGA	508
235	93592	94101	TCGGTGCACGTAGAGCAGCG	CGTGCGTCAGAAAGTCAGTGGGA	509
236	93993	94503	TTTCCCCGAGCTCCGACGCCT	TGGCTTCCACGTTGAGGATCTTG	510
237	94400	94908	ATCCCCCATCACCATCCCCTTCG	TGATGTGGCTTGAGAGCAGCGAC	508
238	94800	95298	GACAACCCCACCACGTACACCAC	TGCGGACGGTGTCGTTGAGCTTA	498
239	95201	95706	GGACCTGACGTTTTTCTTCCCCG	TGACGATACGCGGTGTACACAGC	505
240	95600	96100	CAAGGTGGGCAACATCACGCTCT	GCTGAGATGCGCGTTACCAGACG	500
241	95996	96495	GACGTTGATCGCCGAACACCTGG	GCATCAGGAGAAACGCCGGTCGG	499
242	96399	96904	ATACCCGCCATGACCAACAACCG	TTAATGTCGTCGCTCAACGCCGC	505
243	96812	97311	GCCTAAGACGCTGATCGAGTACA	TTGACGTGTAACCCAGACCCATG	499
244	97183	97698	GCGGCAAATCATGCACGTCAGTT	CGTCAGCTTCGCGATGATCGTAG	515
245	97595	98094	AATCACCAACAACCCGCGCGGAC	AGTTACCAAAGTGCGTCTCGGAC	499
246	98000	98486	GCCGATTCTCAGCACCACCACGC	GCACGTAGCCGCGGGTGTGATCT	486
247	98405	98909	CCCATTCCGCAGCGTCTACATCT	AGGCGGTCTTCATGTCGATCAGC	504
248	98794	99293	CGAGGAGGTAAAGGCTGCCGTGC	CCGATTCCGAAGGTTGGGGTCGA	499
249	99192	99691	CCGATAACAAATCCGCGACCTGC	GTTTGAGACTCGCCGTTCGCTTC	499

250	99602	100112	CGACCTCCGACCCGAAGAATTGC	AGCCGCGTTCTCCGTTACGTTGT	510
251	100000	100500	TACGACGTAAAGCCGCGCATCAC	GTACAAAGTAAGGCAGTGGCGCG	500
252	100378	100892	GTTTCAACGTGACCTGCGACCGC	GATGGAAGCGCCCAGCACCGAGA	514
253	100791	101296	TGCCTGTCAGCAATGACGCACCA	CGACTCCATGCTGCCTGCGCGTA	505
254	101209	101713	GTCTGGTCGCCTGCCTATGTAAG	CAGGTGTCGGTGTTTGCGCTCGG	504
255	101600	102091	AGCCAGGAGCCCATGTCGATCTA	CTCGTGGGGGCGCATGAAGGGTT	491
256	102004	102502	GCTCTTTATGCACGTCACGCTGG	GTCCGATCCGCTGGTCCAGACGT	498
257	102400	102883	CCAGTATCGCATCCAGGGCAAGC	CTGCCATACGCCTTCCAATTCCG	483
258	102788	103291	GCTACGGTTCAGGGTCAGAATCT	CACGTTGGTGATGAGCCGAAGCA	503
259	103201	103702	GTCGGAAGCGGCCGCGATCA	CCAGTTCGGCGTTACGGTCAGTC	501
260	103589	104111	TTTCAGAGTCAGCGACGCGGCGG	TGGTGTCGTGAGTCTGATTGGTC	522
261	103998	104495	CGGACAACGGCTTCCAGCTGTTG	AACATGGCTTCGGACAGGGGGGT	497
262	104401	104892	ACCCACCGAGGAAGAGGAGGAAG	ACAGGGAATGGGAAAAACACGCG	491
263	104798	105294	AGCACCGCTTCCACTTCCAGCAC	CTGACGAGCGGCGGAGGAGAAAC	496
264	105194	105701	AGGCGGCAACTGGCGTTCACCAC	ATGAACCACCGTCCGGATGGGAG	507
265	105597	106099	CCTTACGGCGTTTGTTGTGTCCA	CGCTACGGCTCCTCCAGGTGCTA	502
266	105994	106500	CGAGAAAAAAGCGTCCTTGGG	CCCTTTCGCTCAGACAGCTACGG	506
267	106394	106900	GTCACGCCGACTAGTTGCTTGTC	CGACGGTCTTTTTTGCCTGGGCT	506
268	106809	107315	CGCGCGAAACCAGCTCGGACTTT	TCTTCCAAACGCAGCGAGCTCAG	506
269	107209	107706	CGGGTCCGCACGGGCAAATTCTC	GAAGGCCGCCAGACGAAACAAGC	497
270	107614	108103	GTCGTTTTCCCCGAAGACCTGGC	GTACGGCAGCGGGGAGAAAAGTG	489
271	107984	108503	CCTCGTCCTGTCTCAGCCCAGAG	CCACCAAGGATCTGGATGACTCC	519
272	108398	108913	ATAGCCCGCTTCAACGCCTGCAC	TTCCATCGTAGCCGCTGTGCCCA	515
273	108786	109296	GCGCCCCCAGCAAAAACCACATT	GCTTCGGACCTGCTCATCGGCAT	510
274	109208	109710	GTAACACGCAACACCCAGAGGCA	AGAGCTCCAAACAGTACGGCGAT	502
275	109594	110097	ACACACGATGACGCGGCAGGGTC	GGAAGGTGACCAACGCGGATAGC	503
276	110016	110506	ACAGCGTCCGAACCCAGCAGGTC	CTCCCTCAGCTGTTGCCGCGTCT	490
277	110386	110900	AGTAACGTAGATCGGGCGAGTCG	GCCCGGGAACGTACCTAGCTACT	514
278	110792	111298	AGAGGCGTTCTCGAACATGGTGT	GATGTGCTCATGGACCGGGTGCG	506
279	111195	111708	GACACGCGCCCTTCGAGATCCTC	CTCAGACGCCGACGATCAGTCCG	513
280	111607	112111	AAATCAAAGTCCACCAGGGCGCC	ACGGGTTACAAAAGTCGCGTCTC	504
281	111991	112519	GTCGAATCAGCGTCGTCCCCACG	ACCCGCAAAAAGACATCGAGGCA	528
282	112400	112884	ACCTACAACAGCAGCCTCCGTAA	TCGTCCATGAGGTAAAAGCCCTG	484
283	112785	113301	GGCCACACTTTAACCAGACCTGT	TCGGTGCCGGTCTCTTCTTGTCG	516
284	113211	113705	GTCAAATGTTGGACCGCCGCACG	AGAGGCCCGTTTCTACGATGAAG	494
285	113600	114116	TCAGCCTTCGCGCGCCAAGAACT	TGTGCATGTTGCGCGTTAGTTCG	516

286	114019	114534	TATCTCCTACCCTGTCTCCACCA	GGGGCCCGTACCGTTTTAGAAGA	515
287	114392	114900	TACGCGCTATCGGCCATCATCGG	GAAAATAACAGGACAGTCAGTG	508
288	114785	115316	AACCACAAGGCAGACGGACGGTG	CGCCAGTATCATTACGTTTGG	531
289	115181	115693	CCCGCCAAATACGTCTACTCACA	AGTAGTGACGTTGAGAGCGGTAG	512
290	115583	116099	CGTAAACAAGCGCCAGTCAAAG	ACAGCAGCGAGTCTAGATAGTCC	516
291	116017	116504	CGAAACTACACCTACGTCACC	TCTACTCGCACCTCAACAACCGT	487
292	116401	116909	GTGGAACCGGGTTTGGCACTAGT	CGGTGACGGTAATGGCGACGACG	508
293	116814	117310	CAAACCACATTTACGCTGCCCCA	AGCGTCTGACGGGTACGTAATGA	496
294	117203	117718	AGGCGATATCGGCGTTTTCCCCT	TCTGCGTCGGAGCTCTTTTCATC	515
295	117608	118097	CGACATCGGTTTCACCCACACGC	GTCTCACCGTCGGCGCTACAAGG	489
296	117998	118503	ACGTCGGGGCTGTGTCGCATGAG	TGGTGGAGGTGCAGCGCGTTATG	505
297	118379	118894	TTCCCCCTGAACGACCGCCGGT	CCTGTGGCCGATTACGTGCTGCT	515
298	118792	119281	CCGGGGTGATCTCCAGCTGCTTA	CACGGGCGTGCGGAAGAGAT	489
299	119200	119685	CATCCCCCTCTCGGCGCTTATCG	GCGTTGATCTGCGTGTCCACCTG	485
300	119601	120100	GCGTGTCTGCTTTCGTGGCGTAC	AGACTCGGAAAGCGTGTTGATGC	499
301	120011	120500	CGCATCGACGGCACGCACCT	TGAAGACGGGCAGACGTTCGTTG	489
302	120392	120906	CTGACCGAGACCGTGTGGCTACA	CAGGCGGGGAACGGCGTGATGAT	514
303	120794	121300	GTCCCGGATAACGGCGTTTTTCA	TTCGGTCGAGGGGCAGATTTTGT	506
304	121195	121714	TAACCTGCTTCACCACCACCCGC	GGTGAAACAGCGGCTCCAGAGGC	519
305	121600	122092	TCGCTGTCTCAACTACACGCACC	GAGATGGTATTCGCTGGCGGCTG	492
306	121997	122491	CTGGATTACGAACCCGTGCCACG	TCCGTGATGATTATTGGCTGAGG	494
307	122391	122900	ACCATCACGGCGGCGATTCGACG	GAGCGTTGTACACGGCGAAGATC	509
308	122800	123300	CACCGACTACCTGTTGCACATCC	AAAAATTGGGGCGTACCGGTGAC	500
309	123192	123698	CCTTCTCGCAACCCATGCACCGC	GAGGAGCGGCGGCGATCATTGGA	506
310	123602	124099	GAAAGCAGGACGGACCCAGCAAC	ACACAGCATGTCCTCGTAATCGG	497
311	123997	124508	TACGACGGCAACCCAGCAGCAGC	ACACACGTTCCCCCCCAATCTTG	511
312	124407	124889	TTGGTTCGAATCGAGCTTGGCGT	TCCTCCAAACTCCTCACCATAGC	482
313	124790	125307	TGAGGACGAGGAGACGACGACCG	AGAATCACCGTCACAGCCCTATG	517
314	125184	125683	CACACCTATCTATATACACAC	ACGAGACTGTGAGTACGACGAAC	499
315	125600	126089	TGCCGAGTGTCGTCGACATAAGT	CGAGACCGACGACGTTCCATCTG	489
316	125991	126512	ATGGCTACGAAGGACGGGCGGAC	GAATAAAACTCTAGCTCTCGTC	521
317	126383	126893	GGGAAGGATACGGTTTTAAAGGT	CAGCCAGAACCTCACGCCCGAT	510
318	126778	127291	ACAGTTCGGTGTACATAGCGT	ACCCAAAGTGCGCCCTCTAGTCA	513
319	127177	127698	GCGGTTGTGTTGGATGAACTTGT	ATGCCGTTCTGAGCTCCGACATG	521
320	127610	128088	GCTATTTTTTTACCCGCCTGGAG	GGCTCCACGCGTTTTCCACCCTT	478
321	127999	128466	TCGTGGTCTCCTCGGCTCGT	CTCTCGGTCCGCCTTACCCTGGG	467

322	128397	128917	TTTTTGGGGAGCAGTGGGGGAGG	CGGTCCACCCGCCGCGGAACGGA	520
323	128814	129337	ACCGCGGAACGAGCCTAGGAAC	AACCTATAAAACCCGGCGTGCCC	523
324	129230	129700	TAGGGCGGGAGGCAGGCGAGA	GCGGATTATGGGATGTGGCCTCG	470
325	129609	130114	ACCGGGTCCCCTGGTTTAAC	TCCTGTGGAATTCCGGACATACG	505
326	130006	130510	GCTTCGCTCGCTACGTCATCAGA	TCCGCTTACGTATACAGCCACAC	504
327	130413	130917	ACAAGGGGTGGAGTCTAGGGAGG	CCAAGAATGCGGAAGCCAGCGTG	504
328	130806	131308	TGTGACGTAGAGCCGACGATCGC	CCTATGGGCGCCAAACGTGT	502
329	131201	131699	TCTTCGAGTACGTGTCGGGGGTC	AACCCGTTTTCCCACTCAGTCGC	498
330	131595	132098	CGGTGACGGTAGGACAGAAAA	TGAAGTAGAGAAAAGCCGCGGG	503
331	131994	132502	ATTCACTCAGTGGCGGCGTAGC	CTTCAAGCCGTTGCCCACCACCA	508
332	132375	132912	AGTACGCGTTGGAGGCCTACAC	ACCGCGTCACTCAGCATCAGATG	537
333	132816	133301	AGTCGGTGAAGCTGGCGCCACAT	CATAGCGTTCGTCCTTGAGCGGC	485
334	133195	133718	GGCTATTCAGTTCACCAACGGG	CTCACGTCTCGTCCACCACCC	523
335	133600	134109	TTTGGGCAGCTTCGGTCGGCGTT	ACGGATCTGAAATAGGCGTAGGC	509
336	133998	134516	TCAAGAGCAGCCGCGACGAGGT	GGAATCCCAAGCAGCCCTTAGCC	518
337	134412	134900	AAAACCGACCCATCTACCGCGCG	TGACATGCAACGCAAATTCGGC	488
338	134783	135300	ATCGGCTCGCTGCACGGGTTAAC	TACACGGCCATCACTTCGGGATC	517
339	135196	135683	GCGCCGCGAGAACGTGCTTTTTG	CGCCGCCAGCCTCGTCTTCG	487
340	135600	136076	ACTCCATTTGCTTCGGCGTGCCC	GGAGATGACCGGCTGTGTCTGA	476
341	135994	136515	CGCGCTGGCAAACTCCGACATTC	CGAACTCTGCGTACGATTCTGCG	521
342	136396	136900	TGCGATCCGAACGTGTTACTGGC	CCTCGTAGCATTGGGCGCATGTC	504
343	136813	137290	GAGCTCCTCCGTCGCCTATGC	CCCGGCACAGCTGGTGATACACA	477
344	137220	137695	CTCGGAGGTTATCGTCAAGCGG	CGCCGCCACCGTTATTCCTCTGA	475
345	137600	138079	GACGGGAGGCGATGTCGGGGACG	AGGTAAACTGATCCCCCACGTCC	479
346	138019	138504	CTATACCGCAGCCAATACCACCA	ATCTCTGTCCGGGTGCAGTTCGT	485
347	138389	138900	AGGAGATGTGGATGCACGTGCGG	GAGGAAACCGCAGCACCCAGACA	511
348	138806	139303	TGGATTTGGCCCGCGACGAACAT	AGTGGTGTGGATGTAAGCGTAGC	497
349	139189	139691	GGACGAGGGCATCATGGTAGTCT	AGCTGGTATTGCGATTGGTTCCG	502
350	139582	140107	GACCATCACTACTGCGCGTTCCA	CAGACCGCCTGTGGTTTCAA	525
351	139984	140488	CCCCGTGTTGGATTGTGTGCGTA	TTGGTTGATGGTCACGCAGCTGG	504
352	140400	140900	CCATTCTCTCGGCCATCTACAAC	CCCGCTGCTTGTACGAGTTGAAC	500
353	140779	141321	CGACCCGCTGGAAAACACTGACT	GATGCATCAGACGACGGTGGT	542
354	141212	141690	GTGACGTCGGGCAACACCAAAGA	AAACATAGCGGACCGTGAGAGGC	478
355	141612	142103	GGATATCTAGGTGCTGCATGTGT	GTGGCCCCGAAGAAACGCAACAC	491
356	142006	142520	GACGCCGTGCTCTTCTTCGACTC	TCGCAGTCGACCTCGATATCACA	514
357	142407	142900	GACGCGTTTGGTCATCGATCGGC	GTTGTAACCGGTCACAAAGGC	493

358	142817	143316	ACGTCTACGAGTTCCCTTCCGAA	TACGGCGTCCTGCAGACAGTAAC	499
359	143191	143695	ACTATGGCCGAGCTTTACCTGCG	CGCTGAAAACGCCGACGCTGCTA	504
360	143600	144099	AGATGTCGCCGCCCTTGCAATCT	CTTGTCGAGCAGCATACGGCGCA	499
361	143994	144508	CTCGGTGCTTTCGGAACTGCTCA	AAAGACAGGCCGTCACGTAGTGC	514
362	144400	144913	GTCATCTACGGGGACACGGACAG	TGTGCGGCAGGTTAGATTGACGG	513
363	144786	145300	TATCTTACGCCGCTTGGTGCAGG	GAAAGACGGGCGACAGCACGTTG	514
364	145199	145709	AAGATCCGAGCTACGTGCGCGAG	CGACGTGAGCGAGTCCTTTGAGC	510
365	145597	146109	AGGGAGACCGTATCGCGTAGGAC	TGGTGGCCTTCATCAATCAGACG	512
366	146009	146522	CTGGTTGGTGGAGAGCTTGAGT	TATGTCTAGCGTGAGCGGCGTGC	513
367	146408	146927	GTTCACCGTCGACGCCACTTTGC	GACTCGGACTACCACGGCCTGAG	519
368	146805	147291	CGGGTCGCAGAAAAAGTGCTTGT	AGAAAAAGGAGGAGACGATCCGG	486
369	147186	147712	GGGACATAGGTAAATGGGACCCA	CGGTCTTCGATTTCCACCTGCAC	526
370	147604	148100	CGCACCACGGCCAGATGATTCAG	CTCACCAATGCGACCAACACCAC	496
371	147993	148499	GGCCAGTTCTGCATCTAGGTCGC	GTGAGCAGTAACGGCCCAACGAC	506
372	148384	148900	TGCGGACTGCACAAGGTAGCGGC	CGCCGTGCACGTACTTGAGGAAG	516
373	148800	149305	GCCGCGCGTCTACGATCTGTTGC	AACAGCAGAGGGAGACGGCACAG	505
374	149182	149716	GCCATGCTCAAGACAGACACGGT	GAGGAGGAGGAGGAGGGGGAGAT	534
375	149600	150081	TAAGCAGTCACAGCGGCAGCGGT	CCACGATGACGGTGCCCAACGAT	481
376	149997	150499	GTCGACCCATCACCGTCATCCGC	GCCCAGCTCGCGATTCAGAAAGC	502
377	150415	150905	GCAGCAGCTGGTGCATCACGTGC	GGTATACGGCCAAATCAGCGCGG	490
378	150799	151297	TTTCGGGGTGGACCTTCGGCTGC	GTCCAGATGGCGCTCGCACTTGA	498
379	151194	151704	TCCTCTTTGCACTCAGCGCCGCG	GAAGTGAGGCGCAGGAGACG	510
380	151600	152100	TCACCGGCTGGCTTTCGACGTCA	ACAGGTCCAGCTTAAAGGCGCAC	500
381	151999	152494	AGATCTCAGAGGCCACCCACCCG	CTTTGGCGGGTCCGTCTTTCAAG	495
382	152382	152895	TAGGCGGCGAGGCGAAACTGGTG	CGTTTCCCCACCCGCTTAGAGTT	513
383	152797	153300	CTGTTCGAACGTGGAGGGCGGTC	ATGGTGACCGAGGGGTACACGCA	503
384	153194	153698	GTCGTGTTTGTGTCTGTGGTGGT	TCCTCAAGTACCCGGTGCAAATG	504
385	153597	154085	CGGGGGGCATTTTGTGTGTGTTA	GCTGTAGCGGCGGAGGACTCAAG	488
386	153988	154502	CGGCCAGAAAGTGCGCAATGTCT	GCGGTACACGTCTCTCCAACGAG	514
387	154398	154900	GTCGTTCCAGTGCGTTCAACCCG	CCGGAACCTGCAACAGCAAGCTG	502
388	154794	155293	CAGTTGCCTCTTGATGCTGTGCA	CGACTTCCAGCGGCCCGTCTTCA	499
389	155201	155706	TGATTCAGCAGATCGGCCTCGTC	GTGCTGACGGGTGATGTGGAGCG	505
390	155593	156100	ATGGATCAGGAGTTGCGGACCCA	CGGCAAGCTCACGGTAACTTCTG	507
391	155991	156493	CTTGCATCTTGGTGGTTAACG	CAACGCCGTACTGAGCATGTTCC	502
392	156394	156893	CTGCAACAACAGACTGACGGCGT	ACAACGTACCGCTACTGCAAGAG	499
393	156800	157304	TCGTGGATCTGCGTGAGGCGACC	GTCAACGCGAAGAGTACCACGGC	504

394	157200	157678	GAATTTCGCGTCGGTTCAGGTCG	TCTGGGCCTATCCAAGAAACTGG	478
395	157599	158107	AGTCGTGCATGCGCCGCAGATCC	CAGCGGTAGCAAACACGGAGGCA	508
396	158000	158500	GAAGAAGAGGCCGCCGCTATCGC	GCACAGCTCCGCGGAATGTCACA	500
397	158394	158904	AGAAAGCGGGCGATAGCTGCACG	AACGGCATCATGTTCCTACACGC	510
398	158808	159339	GACGCGCGCCCTCTTCCATGATG	GACGAGCCTGCTGCACCGACATC	531
399	159218	159708	ATTCCCAGGGCGCCTCCAAGAAC	ACCAACCACGCGTTTCGTAGAAC	490
400	159607	160107	CGAACACTCAGCAGGCGCTCGTT	GCTGTGTCGTGTATTGTTGCCCG	500
401	160000	160497	GTCTTAAAAACCGCGGCGTCGAT	GCACTTTGCGGTTCAGTACACGG	497
402	160403	160915	TGTGCAGGTCCCGATACAGGTAC	TGAGTGAGGAAGTTTCGCCGCGC	512
403	160800	161309	TGTCAGATCCAGTAGCCGCTGCA	ACCTACCAGCGGCTCATCTACTG	509
404	161214	161710	CATCAGAAGCACCGTGTCCAGTT	TAAGCCACGCCACGGTACAGACG	496
405	161615	162109	CGTCGTGCTGGTGTCGTAGGAAG	CCTCGAACTCTACCAGGCCCTCG	494
406	161997	162518	GCTCAAAGCCAAACGCGAGGTAA	TCGGGCCCGTCATAGTCATAGAC	521
407	162397	162900	ACGCTCAAAACGGAGGTGTTCGC	TGAGTCTAGATGGCGCCGATAAC	503
408	162798	163331	CTGAGCGGCGCTGACAGCATACG	GGTGCTCAGACCGCTACTCACGC	533
409	163197	163718	AGTGGTGGCGGCGTCGTCAGTTG	ACACAGTGGCGTGCGGCTTTCAC	521
410	163600	164096	TTCAGCTCATCATGCGCCACGGT	CGGCACGCGATCAAAACCCACGG	496
411	164000	164512	GGGCGAGGTGTTGGAATCGGTGT	TCGAAAGCCGACTCCTGACCCAG	512
412	164394	164910	GCTTTCAACGTGCCTAGCGTGTT	CTGACATTAGCGCGTCCAGGC	516
413	164792	165290	GCATCTACCCGACGTGGTTTTGG	CCACAGATGCGCCACCTCGTCGT	498
414	165208	165702	TGCGCGCCGACATGCTCGAGTTC	ATAATACAGCCGCGCCGAGCCAG	494
415	165597	166103	CTTCTGGCCAGGATGCTCAAGTG	GGTCCTTGCTGGAAGTCAGGTAC	506
416	165999	166517	ACAGCTCATGCCTCTACATCAC	TGGTGACGGCGCAATTGAGCAGC	518
417	166399	166909	CGGCAGCGAGCTGGAATTCACGG	GGAAAGACCGTGGTCGTGATCAC	510
418	166819	167322	CAAGAAGCACGACCGCGGTGGTG	GCCTAAAGTGTCCGATCCGCGCG	503
419	167201	167701	CGTGCCACGTGACTCAATTCCGT	TCGCAGCACAGGAAGGTCTTCTC	500
420	167600	168117	CTGGCCGAACGGTTGGTTTTTCA	CGTTCAGCGAGCACGTCTCTT	517
421	168000	168500	TCCATCTTCAGCTGGCGAGACGG	GGGCTCCATGTCGGTGGCAGTGA	500
422	168390	168906	CGTTTGCGCAAAAAGAAGGGGCT	CCGTAGCAGCAATGATGGTAC	516
423	168793	169300	TGGCCGTGGTGTTCACCGTGGTG	CCTGGCCCGAATGACGACGAGAC	507
424	169222	169703	GGAATTCCCCTGCCTACGTCTTC	CGGCGCGTGTTGTTACCCACTTC	481
425	169599	170113	TCGACGTGTACTGCTGCCGCCAG	CACACGTCACGACACCGATCGAT	514
426	169993	170506	ACCCAACGATCATCACTACGCCT	TCACGTCGTCTTGAAACACCGTC	513
427	170404	170900	CATCTTAAGAAACCGGGTCGCCT	CTCGGCGTCGATGGGGTTCACCT	496
428	170800	171310	TAACGGAGGACACAGAGCGGGAG	GTAGAGACCCGAGTCGTGCCGCA	510
429	171195	171699	AGATGGACGTGGGGGACTGATC	CGACGCGCTTCCAGCACCTGCTT	504

430	171587	172107	TTGTTCCCGGTGGAGGTGCGCTC	ACCATCTGTACCACGTCGCCCTC	520
431	172007	172497	GCCTCCGTCGAGAGCGAGTTGTC	TGACGTTAATGTCCGTACCGTGG	490
432	172401	172886	ACGTAGGTGTCTGTGTGGCTGTT	GTTGTCGGTGACGTTGGTTTC	485
433	172793	173300	CCTTACCGGCCGCAGTTACGTAT	TCCCTCGTCCCGACAAATGTAGC	507
434	173186	173701	CATATGTACTGTGGCGGCGGGCA	AGGTGTCTTCAACGACGTGGGGC	515
435	173604	174117	CTTCATCCAGCTCCGTTCGCGCA	GTCGATGGTGTAGGAAGCGTAGC	513
436	174005	174504	GGCCCATGGTGCAGTACGACGAC	TTCCAGACGCACGATGGCCTCAC	499
437	174404	174899	GTTTGGCCGAGGACGTGGTTATG	CAGCGGTCGCAGGACGGGATATC	495
438	174800	175300	CGGGCTCGTTGCACCACTTTGAA	TAGCTACGGCAGCGACGTCGAAG	500
439	175196	175699	TCGTCCTGAGACAGACACGTAGA	TGCCGATGTTTTTCGCCGTCCTG	503
440	175597	176091	GGCAGACGGCAGCGAAACAGAAT	GGGGACGAGGACGAAGAGCAGGA	494
441	175990	176500	GAAGAAAAAGAGCTCGCCCTCGC	CTGCCTCACCTCCATCAAACGCC	510
442	176400	176900	CGTGTCGCAGCTGCAAGTGTCGC	TCTGCATCCTCGCTAACCACTAC	500
443	176805	177300	GCGTCTTAAGCGGCTCCGAGTAC	CCTTCAGTCACCCCAGCCCTTAC	495
444	177189	177698	CAAAACCAGAGCGGAACTTGAG	CGGTGTCGGCAGCCCTGGAATTC	509
445	177601	178110	GAGCTCGTCGTCGTCCTCCTCTC	GACGAGCTGCAGACGGCTGTGTC	509
446	178000	178499	CGCGTAGCTCACCACCTTGTCCT	ACCTATACTATTACCGCCCCACC	499
447	178407	178905	CGGCTCGCAGGACTGAATCGTCG	CCGCGTCCAACGACACATCCACA	498
448	178791	179313	CGACGGGTTCTGGTGCTTTCTGA	TCGTAGCGCTACAGACACCTTAC	522
449	179204	179702	CGTGCCAATGGTGCGTCGCAGTG	CTTGATCGCCGCCGACCGCTATC	498
450	179599	180100	GCCCGGCGAGCCATGTCAATATC	AAGCGCCGCTGACTTGTTTCTTC	501
451	179994	180513	GGCCCCGTCATGTTGCAAGTGTC	CGCAACGCACCTCGAACTCCTC	519
452	180402	180899	CGGCGTACGGTGCTGTTCAACGA	CACGTTCATGATGAGACGGCCCG	497
453	180801	181300	GTGAAACCGCTCGACCTTAACCT	GCCTCGAAGCGGAACGGTTTGTG	499
454	181202	181701	CTCGTCGGTACCCAAGAAACATG	CGCTGGTCGGTTTCTGACTGCC	499
455	181601	182104	CAAGAAACCGGGAACTAGCG	AAGGTAGGTGTCGGGGCCGGA	503
456	181996	182506	TGACGTTCGGCGCCGGAGTTAAC	TGACTGGGAGGCGAAATGACGTC	510
457	182400	182902	GACAGCGAGGAGGAAGACGACGA	GGGGACGGAGAGGCTGACGAAAG	502
458	182797	183299	CTCGACAGCCGTCAGCTTCG	GCTCTTCTGGGGCGACGATAGG	502
459	183192	183709	GCTTTGCAATCACGCCGCTCGAC	CCGCTCAAAGTCACGCTGTGGAC	517
460	183600	184113	TACGAGTACACGTTACCGTCGCC	ACGTGGATTCACTATGCGTGGAC	513
461	183994	184501	AGGAAAAGGCGCGCGGCATGTTG	CAGGCGGTGCAGATCGGTTTTC	507
462	184402	184900	TTCCCGGCGTCGTGAAAGGCAC	ACCTCGTGGATCGAAGCGCTACG	498
463	184792	185298	ACGACTCGTAGGCCGTCAGGTGC	GCGGTGGCGGCGACCAGATTATG	506
464	185190	185706	GTGAAGCTGACGAGCGGCAG	CGCCGTGACACATCAGGTCCATC	516
465	185598	186091	GAGCAGCAGGAGTTCGAGCAAGA	CGGGAGCGATCTACGGAGACCTG	493

466	185993	186505	GCACACACACAAAGTAGTCTCG	ACGTAGATGCACTGCGAGGGCCA	512
467	186385	186899	TTTGCACGCGCGACTTGGCCGAC	GCAAGCCAGCAACTCGCGGAAAG	514
468	186791	187304	GCCCTTGGTGCATCCCGATCATC	CTCGGGATTGGCCTCGTACAGCG	513
469	187198	187698	ACCTCAGGGGCTTCGTGCGCGT	GTGTCGCCCAGCATGATGCCGTG	500
470	187608	188101	GGGTTCACCTGCAGCGCTACGTG	GCCCAGGCGGATCTTGCTAC	493
471	187998	188516	AGCACACCTTCGCGGGCATGTAC	ACAGTCTCCTCCTTCACGACGCC	518
472	188400	188905	CGCGAAACCGGTGAGCAGCCATA	CATGGCGTGCTTGGGGTGTTTGG	505
473	188788	189298	TCGTAATCGACAGGAGGCTGTGC	GCGCTCCTCGGGGTCCAGAAACA	510
474	189205	189709	CACCCATTACTTGCGGCAGCTAT	GTAGTCGTCGACCAGCGTCTGCG	504
475	189593	190102	GCGTTTCACCTGCCGGCGATTCG	GCACAGCAGCTCGCGCTTGATGC	509
476	190000	190518	CGAGGACACGTTCGTGGAGACCT	AATTCAAACCGCCGTCGGGTGC	518
477	190403	190889	GAGGTCGGAGGTCACGCCACATG	GTTGCCGTGACGCAGTCTGCC	486
478	190819	191309	AGCGCGGCCTGCTACACAGCTAC	CGACTATCCTCAACGCGACGAGC	490
479	191207	191702	ACTGTTCCGTCCTGCTGCTTCGT	CGACTACGTGTACTGCGCCCTGC	495
480	191587	192088	GATCACGCACCATAAACTCGCCT	GGCGCGCTCTACCTCTATCGGCA	501
481	192003	192499	ATCTCTTCGACCGAAAGCAGCCG	TTCGCGCTCACCAGCCTCGTC	496
482	192399	192880	TAGTTGATGATCACCGCGTGCGT	GACACCCAAGGCCGGCAAGTC	481
483	192784	193299	TGTTATCGTCGCCACCGTCGCCG	GGAGCCGGCCCTCACTATATAAC	515
484	193202	193700	CGACATCCTCTCGTCTGCTGAG	ACGGCGGTCTTCTTCTGCTGATC	498
485	193600	194113	GCTTGGAGTCACTACCTGTGCTG	AGTGAGGAAAGGGCGCGTTCTCG	513
486	193999	194487	GAAAACAGCGTGGAAGGCGGTCA	GCAAAACACCGCCGTGCTCGA	488
487	194386	194898	CTTCCCGTTTGACTCGCGTGCCA	CGGCCACAAAGGTCAACGAGTCA	512
488	194792	195298	ACACAAGGGCGATCACAACGTGC	CGTGTCATGCCGCGAGTCACAA	506
489	195200	195701	CTTCGTGGACGACTGTATGCGCG	TTCTGCTCTAAAAACACCCCGCC	501
490	195599	196093	CTCTCACGGGAAGGCTAGTTAGA	TCGAAGCGCAGGTAAACGGAATC	494
491	195989	196498	CCACCGTCACGCTGCAAAAACAC	CGCACAGATGGCATGAGTCCTTG	509
492	196398	196903	GTGCGGCGCCTCTCTCATGGATC	GTCTACACTGAACTTGAGCAC	505
493	196783	197320	CCGAAGAACATTGGGACCAGTT	CGTACTTGTTCTCCATGTCCTGC	537
494	197200	197709	CGTGGGCATATCAGAAGTCGGTC	CAACCGCGGTGAGTCTTACCTGA	509
495	197587	198092	CCCGAGACTGTACTTCCACA	ACCTGCGAAAAGAGGAAGTTGCG	505
496	197981	198495	CGGACAGAGCATAACGGCGTTTG	GTTACCGCGTGCCCCCGTCAACA	514
497	198402	198894	GGAATTCGTGGCGATGGACGTCA	ATAAGGTGCCCGAGGATAGCGAG	492
498	198809	199304	GGCTACGTAACATCCCTGATGGA	ATCACAGAGCGTGCTGACGTGGA	495
499	199204	199702	TTAAAGTTTCCCCGTCCCACCCA	GGGGTCAAAACGCGGACTGAAAG	498
500	199585	200100	CCACTCTCAACATCCACAGCACA	GGGTCTCTTTGACACGAGCGCGG	515
501	200000	200498	CATTTCCAACCGACAGTGACG	TGACTAAACTGTACACCAGCCGC	498

502	200390	200905	CGGACGTAGAGGCGCTCGATTA	CTGGGTACTTATGAGGAGCGCGC	515
503	200790	201304	GGGAAGAGGCAGACAAGCAAAGA	CTCATCTGCGGCAGCGGTACC	514
504	201188	201700	GGACGTTTCATTCGCCGAACCGC	TTCATCAGCCAGAGCATCGCGGA	512
505	201600	202106	TCCGTCAGCCAGTAATCCCTCCG	ACGCAGCGGTCAAGTGTTACACA	506
506	201993	202508	GTGATGAGCCGAAACGAGAGG	CCCATCCCCGGTCGCTTATGC	515
507	202381	202889	CGCCCATACCTGTCTGTGTTTCA	CAACGCACACTCAACGTGAAG	508
508	202800	203295	CGTCGTTTCCACTTGCTCGGCTC	ATACGCTTCTCCAACCAACGGTG	495
509	203179	203699	TTGGCGTAGTTGACGGAGTCC	CGCCACTTTGGTCGGGATCGCTC	520
510	203600	204117	GGTGATACGGCGTGATGCTGTGA	AAACGCCAACTCCGTGCCAGCTG	517
511	203999	204518	GGAATCAGGGACGTCGGCTTTGG	CCCACTACGACTACGAGAACGAC	519
512	204385	204898	GGGGTGCTGGATTTTTTGGGACT	TCAGCCTCCATGATGCCTGCATA	513
513	204800	205289	AACGAACTTGATCGCGAGGGGGA	GGGTCTTCCGAAAGGCTTTGC	489
514	205200	205717	ATGAGAATCCATGCGCTGTGTGC	ACCCTCGGTAGTGGACATGGTTA	517
515	205600	206121	TAGGAGGGTCGTAGGTGCAGATA	ACAACACGACACTACCACTCCAC	521
516	205982	206495	CGAGCATACCGCTGTATCTGT	GTACATAGACAAACCAATGGT	513
517	206386	206901	GTACGATTATCTGTGCATTGCC	GCAGTGAACCTCAGCAACGCGAC	515
518	206802	207311	TCCAAGAATAATGCGTTGCCG	ACGTAACAACCATCGTCACGACG	509
519	207205	207720	GGAGAATCCAGCGACGGCACGTA	ACGGTTACACGATTTCGCTGCAG	515
520	207597	208105	TAACGTTCCCTCCCTGTGGCCCG	CACCGTAATGACACCCACGCTGG	508
521	208021	208516	CGCGCTGGAATTTGTCGTCAGTC	TGGGTTTCCAGATACGCGCTG	495
522	208400	208904	CCCGCTGTTCTAGTTACCGTGCT	GGTCACCGGCTTCGTCCTGCTAG	504
523	208809	209319	CGGAGACGGTCTATGCAGAAACG	CCGCGTGGCTGTAGCTATTGTG	510
524	209201	209700	TACACGATACACAGCAAGGCCGG	CTGTGGAATGGAAGTCTCCCGAC	499
525	209607	210096	CCTCGAAATGTAACGTTGCCG	TCCCCGTAAGATGATCCTCGTA	489
526	210000	210501	CTGCATCACGAGGCTGGCATCTG	ACCCCTGGACGGTACAGTTTATG	501
527	210378	210913	TCAAGCTGCTTCGAGACGCTC	CACTCACGCTGGTTGGGCAGTCG	535
528	210822	211303	GTTTGTGTCTCAGAGTTGCCGG	TACAGTAACGCAGGGAGACACAG	481
529	211199	211700	CCGTTCCATCCACAAAGTTCGCA	TGCTGTATGGGCAGGCGTAGTGG	501
530	211604	212100	GGGAAGTTTGGTGTAATGCGGCT	TAACAGGATACGTTGGTAGC	496
531	211994	212488	GCACATTTTATGCGTGGGGTAC	CGTAATCTCTATCATAGCTCGC	494
532	212393	212911	GGCTCGTCGTGCAAAAAACAG	TGTCTTTCAGTCGCCTGGATACG	518
533	212796	213316	TGAGAGGTAAGGATGCGTAGCCA	ACCAACGTTACCTCCGACTTGAG	520
534	213162	213711	GTAGCGTTTGCTGTTACGTTGG	CCTGGGTTTCGGGACTGATGATC	549
535	213600	214101	TCCGTATCCAGGTCCGTCCGCAG	ACACTAGCCATCACAGCGTAGTC	501
536	213985	214503	CACCCTGACGCGTGGTTTCTTTA	AGGAGGAGAAAAACAACACGGGC	518
537	214413	214910	TTTAGCCGCGTGTTGTTCCAGCT	ACAGGGTGTTGAAGGGTAACGTG	497

538	214812	215311	ACACCGTGTTCCACAAGCACCGC	AGAGCACCAGCAATCCCAGGAGG	499
539	215200	215681	GTTTCTCCTCAGCTGCCGTACGT	CAGCGGGGAGGAGAGGAGAGAGA	481
540	215594	216100	TGTTCCATAAAAGCCGGGCGCTC	TTCGGTTTTCTCGTTGGCGGGTC	506
541	216015	216508	TGGACGTCACTTGAGCAGACACA	CCCCAGGGAGAGACTTTCAAC	493
542	216382	216903	CGTTACCTTATGAACCAGCAGC	TGTACCAGCCACAATCACATCCG	521
543	216784	217310	CGTCGCTAGGCTGACAGTTTACG	CGAAGAAGCCAGAATCTCCCGAC	526
544	217183	217690	GTGGTTGTGCAACGTTACGGGTA	TTTAGTACCCCCCCACCCACCAA	507
545	217600	218099	TCGCTGTAGCTGTGGTTCAACCT	TATGATATTTGTTCCCCAGAG	499
546	217983	218535	GAAGATGGTGAACTGTGGAGAG	AAGTCACCGCCGTTCTCCCCATC	552
547	218421	218933	GGTTTAGCGGCTTCCCCAGATCG	TCCGAGATTAAGGAAAGGAGAA	512
548	218818	219308	GCCACCGCGTTATCCATTCCTCG	GTTTGGCTGCTGACCGACGCGTC	490
549	219208	219710	CGAGGACCGCGATCTCCGTATAG	GACCCGGGGATCTTGGATTCACC	502
550	219609	220100	CGAAGAATGAAAGACGACGATG	AGGCGGCTTTGAGAGCGAAGAAG	491
551	220019	220500	TCAGCGTTGCAGTAGTCGACTCC	CGCGGTGTCGTGACAGGAGAGTG	481
552	220401	220883	CACCAAAGAAGATTACCGTCC	TGTGTTGCTACAGGGAGTGAAGG	482
553	220793	221300	CGGTCGCACGGATTATTCCTTCT	TGCTACGACTCGCCCACATCACA	507
554	221195	221739	GGTCGTTTCGGCGTGAAGTTGGA	ACAACATTGGCGAAGGCGCAGCG	544
555	221602	222091	CCATGGTTCCGAAGCGTCCCCAC	TCACCCTCGCCTATTTAACCTCC	489
556	222000	222500	GGCGTGGTGTGGGTGCTGTTTGT	CCTTTGTACACACTACCGGTGCG	500
557	222391	222904	TCGAAACACGCCGCAAACGCCGC	CAGACGAAGTACGCCGCAACCCG	513
558	222790	223303	GCGGCCGGAGGGAACAACAAGTG	CACCTAGGTGGACGGCCGACATC	513
559	223197	223715	CACGCGGCTCCAAGACTCCAAAT	CGCTGTTGACGTCGCTGCCATCG	518
560	223587	224110	ATGGCCCAGCGCAACGGCATGTC	ACCAACACGATCCACGCGTCACG	523
561	224009	224509	GTACGGATTCTACGCGCGGGTGA	AGCACCATCGGCTGCAGTCG	500
562	224389	224897	TCCGACAGACGTTGCTGCGC	GTGCGCGTCCGCGTTTGGCA	508
563	224808	225310	GTGGGGTCCGCTGTACCTGT	TTTATGGGGTCACCGCGTTGTTC	502
564	225178	225696	AGCTGGACCGCGAGCAGTGGGA	TTTCGGCGCCTCGTCGTTGAGTG	518
565	225590	226094	AGCCTGGTTGGATCTCGACGAAT	CCTGTGGCCCGAAGATGTACA	504
566	226019	226519	GGATAGAACCGCGGCCCATCTCT	CATCCTGCGTCGGAGCTCAGACC	500
567	226391	226889	TAGCGCGTGTCCCTGCCAGTCTC	GGTTGGGAGATGCGGGGTGGTGC	498
A1	192000	192500	CCAGACAAACCTCCGCGCTTATG	AAGCTCCTCGTCATCCTCCGTGT	500
A2	192419	192909	GGGTCTTTTCACGCGTAGCCTCC	GCGTGCTGTTTTCCGTGTTGCCG	490
A3	192800	193294	CTCTTCTTCCTCTCCGTCGCTGT	ACACGTCTAGCGCGTACATGTGG	494
A4	193215	193706	CACTATTGTCCAGGCCACATGCG	CTCTGGAAAGCCTGGGTGGGTCT	491
A5	193590	194097	ATGCTTGGCCGTGGACTTCCAGG	CTGGGCTGCAAGGTACGGACTCT	507
A6	193985	194492	AGCAGCCATAGCACCAGAGTCCC	GTGAACGTGGGTATCGTCTCTC	507

A7	194400	194909	CTCGAAATAGGCTCCGCGCTCAT	AAACCGCCGTGTGGAAAGTCCCG	509
A8	194802	195310	CTTGGTGACGTTGTTTCCCCT	GTCGCGATAGACAACTCCGGTCG	508
A9	195209	195700	GCTACACCTCTCTGTCGCGACAT	AACGCACAAATCCCGTCCGAACG	491
A10	195585	196118	GCGTTAACAACCGGTGGCTAGGA	TCTCGTCAGGCTTGTCATGGTCT	533
A11	196001	196506	CTCAACAATACCGCCCTACGT	GGTTACGCTGCTACAGTACTGC	505
A12	196383	196888	GACAGGATAGGTCAAAAGAT	TCGTGGTATCTCACACCGTCAGC	505
A13	196816	197296	TGTCCCTCAAACCGCCAAACGCT	GGCACGCCTGTGTACCCCAAAAG	480
A14	197155	197700	CCCGTTCCCCACACAAATGACGA	GTGGCGTGTACAGGGTACGTTCT	545
A15	197607	198114	GTGCAGACGGCCCCACTTTCGT	TCCTCTATGTGGTACTGGTGCAG	507
A16	198004	198500	GACCTCGGACCCAGGGACGTATC	GTTTGGGGACGTGTCCGGCTACC	496
A17	198401	198906	GAAGTTGCCACGCACAAGCCAGG	TCATACTGACGTGAGGCGCTCCG	505
A18	198792	199300	CGCGAGTATTACGTGTCAGGGGA	CTGCTACCCTAACACTCGCCTCC	508
A19	199184	199702	CGCCTAGATACCAGAGGTACG	GAAGCGGAGTGCATCTCGAGTCG	518
A20	199598	200099	AGATCGCCAGAGGGTTCCGGAGG	CTGGAGGGCTACGCGGATCGAGT	501
A21	200006	200501	GAACACGATAGTCCCGGAGCCAG	CCCCGCACCCTAAAACACCGTGT	495
A22	200378	200903	GGCTGTTACCCTGTCTAGACTCA	GGCCTGGAACATGCAACGACGGT	525
A23	200803	201303	GGTATGGTTCCCAGGACTGCACG	TCCGTCGAGTCACCGTAATCACC	500
A24	201200	201706	GCTGGTCTGGAACACGACAGTGC	GCTACGGCGCCATCGTCTTCCTT	506
A25	201593	202111	CTACGTAGATCAGCAGCGAGCCG	TCTGCGGAATGATTTGGCTGGG	518
A26	202007	202500	CCAAACACCAGAGAAGGGAGGGG	TAATCTATCACACCACACTCCTG	493
